# Impact of delayed ventricular wall area ratio on pathophysiology of mechanical dyssynchrony: implication from single-ventricle physiology and 0D modeling

**DOI:** 10.1186/s12576-020-00765-y

**Published:** 2020-08-06

**Authors:** Yohsuke Hayama, Shuji Shimizu, Toru Kawada, Jun Negishi, Heima Sakaguchi, Aya Miyazaki, Hideo Ohuchi, Osamu Yamada, Kenichi Kurosaki, Masaru Sugimachi

**Affiliations:** 1 Department of Cardiovascular Dynamics, National Cerebral and Cardiovascular Center, 6-1, Kishibe-shimmachi, Suita, Osaka 564-8565 Japan; 2 Department of Cardiovascular Science, Faculty of Medicine, Osaka University Graduate School of Medicine, 2-2, Yamada-oka, Suita, Osaka 565-0871 Japan; 3 Department of Pediatric Cardiology, National Cerebral and Cardiovascular Center, 6-1, Kishibe-shimmachi, Suita, Osaka 564-8565 Japan; 4 Department of Cardiology, Shizuoka Children’s Hospital, 860, Urushiyama, Aoi-ku, Shizuoka, Shizuoka 420-8660 Japan

**Keywords:** Dyssynchrony, Mechanical discoordination, Heart failure, Single ventricle, Hemodynamic modeling

## Abstract

Electrical disparity can induce inefficient cardiac performance, representing an uncoordinated wall motion at an earlier activated ventricular wall: an early shortening followed by a systolic rebound stretch. Although regional contractility and distensibility modulate this pathological motion, the effect of a morphological factor has not been emphasized. Our strain analysis in 62 patients with single ventricle revealed that those with an activation delay in 60–70% of ventricular wall area suffered from cardiac dysfunction and mechanical discoordination along with prolonged QRS duration. A computational simulation with a two-compartment ventricular model also suggested that the ventricle with an activation delay in 70% of the total volume was most vulnerable to a large activation delay, accompanied by an uncoordinated motion at an earlier activated wall. Taken together, the ratio of the delayed ventricular wall has a significant impact on the pathophysiology due to an activation delay, potentially highlighting an indicator of cardiac dysfunction.

## Background

Electrically and mechanically synchronous wall motion is essential for efficient cardiac performance. Dyssynchronous contraction and relaxation reduce the cardiac output, eventually leading to adverse myocardial remodeling and heart failure [1]. The disparity of electrical activation causes not only a time delay in regional peak contraction, but also back-and-forth shortening and stretching of the regional myocardium, termed “mechanical discoordination”. A mechanistic understanding of this mechanical discoordination helps provide appropriate indications for cardiac resynchronization therapy [2].

In an anatomically normal left ventricle, the left bundle branch block (LBBB) can induce an uncoordinated septal motion known as a septal flash, which is characterized by an early shortening during the isovolumic contraction period followed by a rebound stretch during the ejection period. Patients with LBBB have a worse prognosis than those with non-specific intraventricular conduction delay or with right bundle branch block [3, 4], which indicates that the uncoordinated motion of the regional myocardium is a key indicator of heart failure associated with mechanical dyssynchrony [5–7]. Regional contractility [8] and diastolic property [9] were reported to modulate the pathophysiology of mechanical discoordination.

Besides the anatomically normal left ventricle, we have reported that a characteristic motion similar to the septal flash could be detected at the earlier activated ventricular wall in pediatric patients with functional single ventricle [10]. In that study, however, the effect of morphological variations on ventricular function was not fully examined. Patients with single ventricle show diverse ventricular morphologies due to developmental abnormality [11], resulting in various ratios of a delayed ventricular wall to an earlier activated wall. Although morphological characteristics associated with spatial disparities in the activation onset of the regional myocardium may play an important role in the pathophysiology of mechanical discoordination [12], no systematic assessment has been performed to examine the effect of a morphological factor on ventricular dysfunction caused by an activation delay. Detailed analysis of clinical data obtained from patients with single ventricle will provide a better understanding of the pathophysiology regarding the relationship between morphological characteristics and uncoordinated wall motions.

In this study, we hypothesized that the ratio of the ventricular wall exhibiting delayed contraction has a pivotal impact on ventricular dysfunction because of the widened time delay of activation onsets. To test this hypothesis, we analyzed ventricular strain in patients with single ventricle with prolonged QRS duration. Further, we developed a two-compartment ventricular model in a zero-dimensional platform, which allows simple characterization of cardiac mechanics, taking no account of pressure and flow wave dynamics across ventricles and vascular trees afforded by higher dimensional model. The hemodynamic simulation study was performed to obtain a deeper understanding of the results of the clinical study.

## Methods

### Subjects

The clinical study complied with the principles outlined in the Declaration of Helsinki and was approved by the institutional review board (M23-002, M28-018). A total of 62 consecutive patients with single-ventricle anatomy were retrospectively identified and included according to the following inclusion criteria: a postoperative state after bidirectional Glenn or Fontan operation, the presence of conduction disturbance defined as QRS duration Z-score ≥ 2 [13, 14], and availability of echocardiograms suitable for strain analysis and cardiac catheterization for hemodynamic analysis which had been performed within a week apart. The exclusion criteria were poor echocardiographic images, complete absence of the ventricular septum, and decline to provide consent under the opt-out method.

### Cardiac catheterization

All patients underwent diagnostic cardiac catheterization with angiography under mild procedural sedation as previously described [15]. Central venous pressure measured at the superior vena cava, ventricular pressure, and aortic pressures were obtained using fluid-filled pressure transducers. Ventricular volume was calculated using Simpson’s method from biplane angiogram and indexed for body surface area (ESVi, ml m^−2^). We estimated ventricular contractility by ventricular elastance at end-systole (estEes, mmHg ml^−1^ m^2^) using the following equation:

1 $${\text{estEes}} = \frac{{{\text{AP}}_{\text{dic}} }}{\text{ESVi}},$$

where AP_dic_ is the dicrotic notch pressure at the ascending aorta.

### Echocardiography and strain analyses

All echocardiograms were obtained between April 2013 and March 2017 using a Philips iE33 ultrasound system (Philips Medical Systems, Andover, MA, USA) by a single observer (Y.H.). The frame rates were maximized at 52–100 frames/s to provide the highest possible temporal resolution. The timings of the atrioventricular and aortic valve opening and closure from QRS onset were measured from pulse-wave Doppler flow sampled in the ventricular inflow or outflow tract and served as cardiac event timing markers. An off-line myocardial strain analysis was performed based on two-dimensional speckle tracking using QLAB 9.1 software (Philips Medical Systems, Andover, MA, USA).

**Fig. 1:**
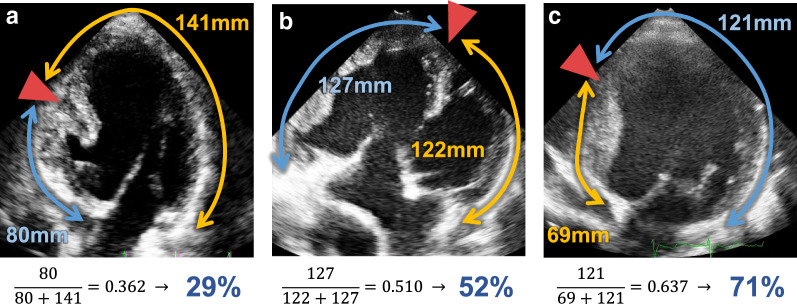
Strategy for estimating the ratio of the delayed wall. From apical four chamber-like views in patients with single ventricle, an insertion point of the septum (red triangles), which separates a ventricular wall into two regions, can be detected. Representative cases of the ventricle with large ventricular septal defect and a hypoplastic chamber, the codominant-type ventricle, and the ventricle with intact ventricular septum and a hypoplastic chamber of closed cavity outside of the systemic ventricle are shown in Panels a, b, and c, respectively. Using the two-dimensional speckle tracking echocardiography, we can judge a wall region that exhibits a relatively delayed contraction by comparing the times to peak strain between the two regions. From longitudinal lengths of the earlier activated and delayed ventricular wall regions (yellow and blue lines, respectively), the length ratio of the delayed region is calculated (formulas shown at the bottom left of each panel). The length ratio is converted into the area ratio (blue percentages shown at the bottom right of each panels) (see Additional file 1: Appendix A)

We assessed the ratio of the ventricular wall exhibiting delayed contraction as follows (Fig. 1). From an apical four chamber-like view, an insertion point of the septum, which separates a ventricular wall into two regions, is detected. As for patients with large ventricular septal defect (Fig. 1a, b), because the bundle of His proceeds along with the rudimentary septum and branches into the apex-forming dominant ventricle and the non-dominant chamber wall [16], conduction disturbance at a branch can induce the delay of an activation onset between the two walls. By comparing the times to peak strain between the two walls, we judged a wall that exhibited a relatively delayed contraction compared with the other wall and denoted it as a delayed wall [10]. As for patients with intact ventricular septum (Fig. 1c), the free wall with the apical cap was consequently judged as a delayed wall compared with the septum in all cases. A length ratio of the delayed wall (LR_delayed_) is calculated as the length of the delayed wall divided by the global base-to-base longitudinal length. An area ratio of the delayed wall (AR_delayed_) was then derived from LR_delayed_ as follows (see Additional file 1: Appendix A):

2 $${\text{AR}}_{\text{delayed}} = \frac{{1 - \cos \left( {\pi \cdot {\text{LR}}_{\text{delayed}} } \right)}}{2} .$$

The extent of an uncoordinated wall motion, which is characterized by a dyskinetic dilation during the ejection period at an earlier activated ventricular wall, was quantified by the strain ratio (R_strains_) derived from two strain values (%) during the isovolumic contraction period and ejection periods; R_strains_ = (100 + Strain_ejection_)/(100 + Strain_isovolumic_) [10]. This index increases if the earlier activated ventricular wall is forcedly dilated and fails to shorten after the delayed ventricular wall starts contraction. The R_strains_ was measured based on a hemiglobal longitudinal strain at three segments on the earlier activated side among seven segments of the global ventricular wall (see Fig. 7a for a representative picture).

### Plasma B-type natriuretic peptide levels

All patients underwent blood sampling after at least 15 min of supine rest, and the plasma level of B-type natriuretic peptide (BNP) was measured and used as an index of ventricular mechanical stress. This surrogate marker was reported to be an independent predictor of all-cause mortality in patients with single ventricle [17].

### Hemodynamic simulation of single-ventricle circulation with a lumped-parameter model

To obtain a deeper understanding of the results of the clinical study, we performed a hemodynamic simulation of Fontan circulation utilizing a previously developed lumped-parameter model of the cardiovascular system with some modifications (detailed in Additional file 1: Appendix B) on Matlab 9.2 and Simulink 8.9 software (MathWorks, Natick, MA, USA) [18–20]. In brief, the cardiovascular system was constructed with a time-varying elastance cardiac chamber model and a three-element Windkessel vascular model. The systemic and pulmonary circuits were connected in series to achieve the postoperative state after total cavopulmonary connection (Fontan operation). The ventricle was designed as a hemisphere consisting of two compartments in which AR_delayed_ was varied. The volume ratio of the delayed compartment was equal to AR_delayed_ under the assumption of identical curvatures (detailed in Additional file 1: Appendix B).

### Simulation of mechanical dyssynchrony with two-compartment model

We specified a time delay of the activation onset at a delayed ventricular wall by Δ*T* (ms). An atrioventricular interval was fixed, whereas Δ*T* varied from 0 to 90 ms in steps of 5 ms. The Δ*T* of 90 ms corresponds to 10 standard deviations of the QRS elongation observed in our patient study. We also varied the volume of the delayed compartment in the two-compartment model from 5 to 95% of the total end-diastolic ventricular volume in steps of 5%. Corresponding to the increase in the ratio of the delayed compartment, the volume in the earlier activated (normal) compartment would decrease from 95 to 5% of the total end-diastolic ventricular volume. In the setting of the ventricle separated into 20 units, ventricular parameters were thus given for one unit (= 5% of the total ventricle). Cardiac and vascular characteristics of the simulation study were tuned to yield typical postoperative hemodynamics of a 40-kg body after Fontan operation, which was comparable to the hemodynamic data at our institute [21] and this patient study.

Our simulation provided two volumetric values of earlier activated and delayed ventricular compartments as a function of time. When earlier activated and delayed ventricular compartments are connected with a sagittal plane perpendicular to the base of the hemisphere, a longitudinal strain can be measured using a coronal cut-plane through the vertex of the hemisphere. This view is analogous to an apical four chamber-like view in the anatomy with single ventricle that includes earlier activated and delayed walls (Fig. 1). In accordance with clinical echocardiography, the longitudinal length of the ventricular hemisphere was divided into seven segments. We estimated two hemiglobal longitudinal strains: one for the three segments on the earlier activated side and the other for three segments on the delayed side. The apical cap segment was excluded to assess the regional strains according to recommendation [22] (detailed in Additional file 1: Appendix C).

### Statistical analyses

All statistical analyses were performed using JMP 12.0.1 (SAS Institute Inc., Cary, NC, USA). Categorical variables were expressed as frequencies and percentages, whereas continuous variables are expressed as the median and interquartile range. Spearman’s correlation analysis was used to assume relationships between continuous variables. Statistical significance was accepted at *p* value < 0.05. To analyze the combined effects of AR_delayed_ and an activation delay in clinical study, we drew a response surface for plasma BNP levels or estEes using a second-order polynomial model.

## Results

### Study population

Subject characteristics in the patient study are summarized in Table 1. Among a total of 62 patients, 51 (82%) had large ventricular septal defects, and 42 (68%) exhibited delayed activation at the free wall of the apex-forming dominant ventricle.

**Table 1:** Subject characteristics in the patient study

Variables	Values
*n*	62
Male, *n*	38 (61)
Age, year	14.2 [4.9–24.5]
Weight, kg	40.0 [13.5–49.2]
NYHA functional class (I/II/III), *n*	35 (56)/19 (31)/8 (13)
Final palliation (Fontan/Glenn), *n*	51 (82)/11 (18)
QRS duration (Z-score)	3.8 [2.5–6.2]
Anatomy	
Dominant ventricle (= morphological left ventricle), *n*	22 (35)
Presence of ventricular septal defect, *n*	51 (82)
Delayed ventricular wall (= including the apex), *n*	42 (68)
Hemodynamics	
Heart rate, beats min^−1^	83 [73–94]
Central venous pressure, mmHg	11 [9–12]
Systolic ventricular pressure, mmHg	101 [95–114]
End-diastolic ventricular pressure, mmHg	8 [6–10]
End-diastolic ventricular volume, ml m^−2^	124 [80–162]
End-systolic ventricular volume, ml m^−2^	56 [38–93]
Ejection fraction, %	50 [39–57]

Values are expressed as median [interquartile range] or as number (percentage). NYHA, New York Heart Association. Primary diagnoses: double-inlet left ventricle 8; double-inlet right ventricle 15; mitral atresia 11; tricuspid atresia 12; unbalanced atrioventricular septal defect 10; hypoplastic left heart syndrome 6

### Relations of area ratio of delayed wall with severity of heart failure

**Fig. 2:**
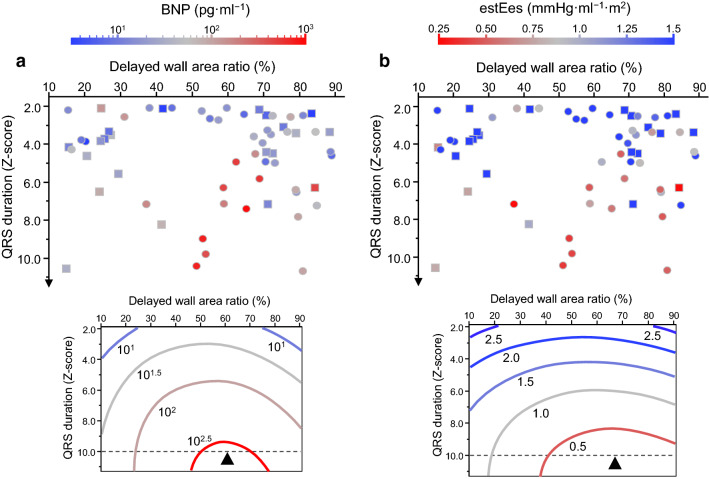
Relationships among QRS duration, the ratio of the delayed wall, and ventricular function. Dot distribution maps in the upper row show clinical profiles of each patient (one dot); colors were according to plasma B-type natriuretic peptide levels (BNP, **a**) and estimated ventricular contractility (estEes, **b**) in the graph fields with QRS duration Z-score on the vertical axis and the ratio of the delayed wall on the horizontal axis. Square and circle dots represent the morphology of the dominant ventricle as the left and right ventricles, respectively. Contour plots in the lower row exhibits iso-response lines of BNP and estEes in response surface methodology as shown in Table 2. The highest BNP and lowest estEes at 10 standard deviations of QRS duration (black triangles) were located at the ratio of the delayed wall of 62% and 68%, respectively

We assessed the relationships among QRS duration, AR_delayed_, and ventricular function evaluated by plasma BNP levels or estEes in this cohort. Figure 2 represents the distribution of patients with colors corresponding to the plasma BNP levels or estEes on the fields with the QRS duration Z-score value as an index of an activation delay on the vertical axis and AR_delayed_ on the horizontal axis. The relationships in the response surface were evaluated using a second-order polynomial model. As shown in Table 2, the BNP levels exhibited a positive correlation with QRS duration Z-score (β = 0.72, p < 0.001) and an inverse U-shaped correlation with AR_delayed_ (β = − 0.31, p < 0.011). Similarly, the estEes showed a negative association with QRS duration Z-score (β = − 0.64, p < 0.001) and a U-shaped association with AR_delayed_ (β = 0.16, p = 0.19), although the association between the estEes and a quadratic term of AR_delayed_ was not statistically significant. According to contour plots exhibiting iso-response lines of BNP and estEes shown at the bottom row in Fig. 2, the highest BNP and the lowest estEes at 10 standard deviations of the QRS duration were located at AR_delayed_ of 62% and 68%, respectively (Fig. 2, bottom).

**Table 2:** Second-order polynomial models by response surface method

	β	*p*
Response variable: log_10_BNP	(*R*^2^ = 0.45)	< .001
Explanatory variable		
QRS duration Z-score (QRSZ)	0.72	< .001
Area ratio of the delayed wall (AR_delayed_)	− 0.122	0.32
(QRSZ − 4.56)^2^	− 0.17	0.24
(AR_delayed_ − 57.2)^2^	− 0.31	0.011
(QRSZ − 4.56) · (AR_delayed_ − 57.2)	0.09	0.41
Response variable: estEes	(*R*^2^ = 0.32)	< .001
Explanatory variable		
QRS duration Z-score (QRSZ)	− 0.64	< .001
Area ratio of the delayed wall (AR_delayed_)	0.00	0.98
(QRSZ − 4.56)^2^	0.15	0.36
(AR_delayed_ − 57.2)^2^	0.16	0.19
(QRSZ − 4.56) · (AR_delayed_ − 57.2)	− 0.06	0.58

β, standardized coefficient; BNP, B-type natriuretic peptide; estEes, estimated ventricular elastance at end-systole; Constant terms 4.56 and 57.2 are means of QRS duration Z-score and the area ratio of the delayed wall in percentage, respectively

### Simulated effects of volume ratio of delayed compartment on mechanical discoordination

**Fig. 3:**
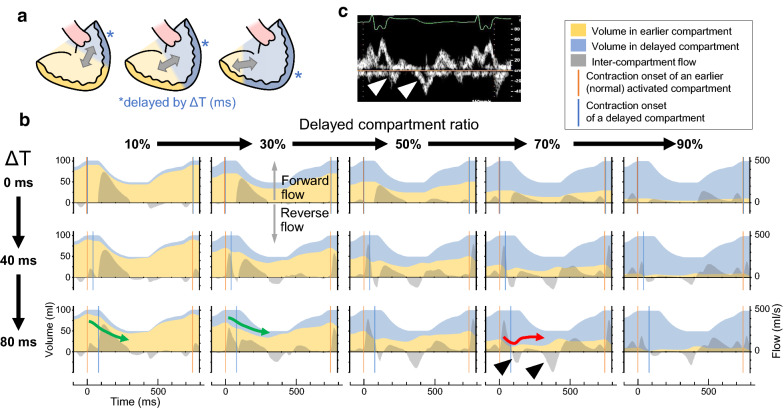
Simulation of mechanical dyssynchrony in a two-compartment ventricular model. Ventricles in a two-compartment ventricular model are schematized in **a**. Delayed activation by Δ*T* ms was applied to a part of the ventricular volume corresponding to a part of the wall area (colored in blue) associated with the ventricular outflow. Gray double arrows represent an inter-compartment flow (clinically called as intraventricular flow). Time–volume relations during a cardiac cycle with an activation delay (Δ*T*, ms) are shown in **b**. Orange and blue vertical lines, yellow and blue areas, or superimposed gray areas represent contraction onsets, ventricular volume changes at every moment of earlier and delayed compartment, or inter-compartment flow, respectively. Both the activation delay by Δ*T* (top to bottom row) and the ratio of the delayed compartment affect ventricular mechanics. A monophasic contraction of the earlier activated compartment with relatively large volume (green arrows) is noted when the ratio of the delayed compartment is 10% or 30%. A preceding volume reduction followed by a systolic stretch (red arrow) can be detected when the ratio of the delayed compartment is 70%, at which ratio an inter-compartment reverse flow is noted in early and late systole (black triangles). This swinging flow is similar to representative Doppler velocity signal at ventricular septal defect in a patient with codominant-type single ventricle (white triangles, **c**)

To obtain a deeper understanding of the results in the clinical study, we performed a hemodynamic simulation using a two-compartment ventricular model. Parameters used in the simulation and modeled hemodynamics at baseline (Δ*T* = 0 ms) are summarized in Tables 3 and 4, respectively. As schematized in Fig. 3a, a delayed compartment (colored in blue), which has the ventricular outflow, constitutes a varied ratio of the total end-diastolic ventricular volume. Both the activation delay by Δ*T* and the ratio of the delayed compartment affect the ventricular mechanics (Fig. 3b). A monophasic contraction of the earlier activated compartment (colored in yellow) with a relatively large volume is observed when the ratio of the delayed compartment is 10% or 30% (green arrows). In contrast, a preceding volume reduction followed by a systolic stretch is detected when the ratio of the delayed compartment is at 70% (the red arrow). Under this situation, inter-compartment reverse flow shows two peaks in early and late systole (black triangular arrow-heads). This swinging flow was similar to a representative Doppler velocity signal at the ventricular septal defect observed in a patient with single ventricle (Fig. 3c, white triangular arrow-heads), assuring the clinical compatibility of our model. A change in the simulated ventricular pressure waveform also represented similarity to the clinical pathophysiology (Additional file 1: Figure S4).

**Table 3:** Parameters used in the simulation

Cardiac parameters	Atrium	Ventricle (per unit)
Maximal elastance (E_max_), mmHg/ml	1.00	62.0^*^
Time to maximal elastance (T_max_), ms	120	290
Scaling factor of EDPVR (A), mmHg	0.80	0.28
Exponent for EDPVR (B), ml^−1^	0.08	0.80^*^
Unstressed volume (V_0_), ml	10.0	0.77^†^

Systemic circulation	Artery	Vein
Characteristic impedance (Z), mmHg∙s∙ml^−1^	0.04	–
Vascular compliance (C), ml/mmHg	1.10	35.0
Vascular resistance (R), mmHg∙s∙ml^−1^	1.05	0.050

Pulmonary circulation	Artery	Vein
Characteristic impedance (Z), mmHg∙s∙ml^−1^	0.01	–
Vascular compliance (C), ml/mmHg	2.5	3.0
Vascular resistance (R), mmHg∙s∙ml^−1^	0.040	0.010

Atrioventricular valve characteristics		
Effective orifice area, cm^2^	5.0	
Inertance (L), mmHg s^2^ ml^−1^	0.0003	
Density of blood (ρ), g ml^−1^	1.06	
Constant (K), g s^−2^ cm^−1^ mmHg^−1^	1 333	

Aortic valve characteristics		
Resistance (R), mmHg∙s∙ml^−1^	0.005	

Intraventricular communication		
Resistance (R), mmHg∙s∙ml^−1^	0.008	

Other parameters		
Heart rate (HR), beats min^−1^	80	
PR interval, ms	100	
Total stressed blood volume, ml	720	

The ventricle was modeled to be separated into 20 units and ventricular parameters were given for one unit (= 5% of the total ventricle). ^*^20 times larger than values for the total ventricle. ^†^20 times smaller than values for the total ventricle. EDPVR, end-diastolic pressure–volume relationship

**Table 4:** Baseline status in the simulation

Modeled patient’s characteristics	
Heart rate, beats min^−1^	80
Body weight, kg	40.0
Body surface area, m^2^	1.35
Hemodynamics	
Mean central venous pressure, mmHg	10.0
Mean atrial pressure, mmHg	5.8
Ventricular end-systolic pressure, mmHg	109
Ventricular end-diastolic pressure, mmHg	8.7
Arterial pressure, mmHg	108/70 (90)
Ventricular end-diastolic volume, ml	102.0
Ejection fraction, %	52
Cardiac index, L min^−1^ m^−2^	3.15
Pulmonary vascular resistance index, units m^2^	1.35
Systemic vascular resistance index, units m^2^	25.6

**Fig. 4:**
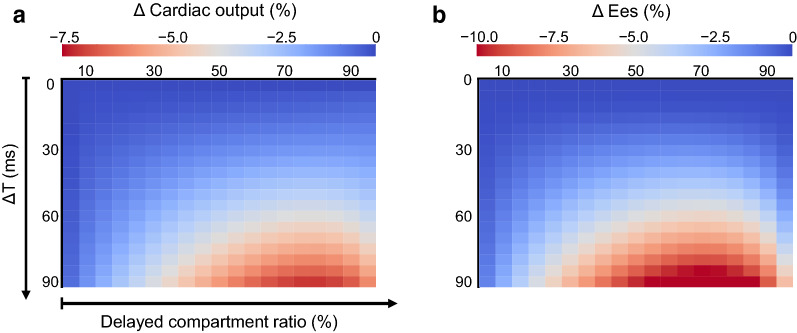
Impact of the ratio of delayed compartment on simulated ventricular dysfunction due to activation delay. Tiled heat maps display the relative change in simulated cardiac output (**a**) and ventricular contractility (Ees) (**b**) with respect to each baseline value (Δ*T* = 0) in response to an activation delay (Δ*T*, ms) and the ratio of the delayed compartment (%). When the delayed compartment constitutes approximately 70% of the total volume, large activation delay can induce large reduction of cardiac output and ventricular contractility

Relative changes in simulated cardiac output and ventricular contractility (ΔEes) with respect to each baseline value (Δ*T* = 0) are shown in Fig. 4. When the delayed compartment constitutes 70% of the total volume, a large activation delay can induce large reductions of cardiac output and ventricular contractility in our simulation.

### Simulated hemiglobal longitudinal strain and hemodynamics

**Fig. 5:**
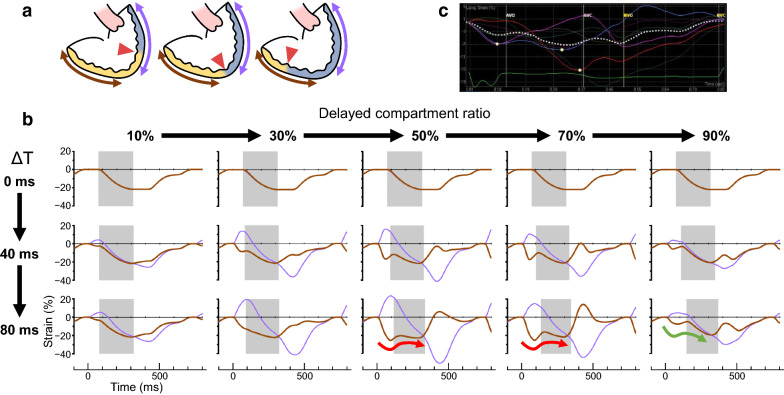
Comparison of hemiglobal strain patterns depending on activation delay and ratio of the delayed compartment. **a** Schemata of a two-compartment ventricular model. Hemiglobal longitudinal strains on the earlier activated and delayed wall regions (brown and purple double arrows, respectively) can be estimated according to Additional file 1: Appendix C. The simulated strains were calculated over hemiglobal three segments among seven global segments without an apical cap segment, irrespective of boundaries (red triangles) between the earlier activated and delayed compartments (walls colored in yellow and blue, respectively). **b** Simulated time–strain curves during a cardiac cycle. The brown and purple lines represent hemiglobal strains on an earlier activated and delayed regions, respectively. The superimposed gray areas indicate the ejection period. As an activation delay of Δ*T* (top to bottom row) and the ratio of the delayed compartment (left to right column) increased, an uncoordinated double-peak shortening was exaggerated at an earlier activated hemiglobal wall (red arrows). When the ratio of the delayed compartment was large enough, a first peak became small (green arrow). **c** A representative hemiglobal longitudinal strain curve at an earlier activated ventricular wall (white dotted line) in a patient with single ventricle. The blue, red, or purple lines represent regional myocardial strain curves, and bottom green line presents the electrocardiogram

Figure 5 illustrates several patterns of the hemiglobal strain curves calculated based on a two-compartment model. As an activation delay of Δ*T* and the ratio of the delayed compartment increased, an uncoordinated double-peaked shortening was exaggerated in the earlier activated hemiglobal strain curve (the red arrows). However, when the ratio of the delayed compartment was large enough, the first peak became small (green arrow). A similar uncoordinated double-peaked shortening was also observed at an earlier activated ventricular wall in a patient with single ventricle (Fig. 5c, white dotted line). As described in the methods, an extent of a dyskinetic dilation during the ejection period at an earlier activated ventricular wall was quantified by the ratio of the two peak strains (R_strains_), which can be measured both in clinical strain measurements and in our simulated strain curves.

**Fig. 6:**
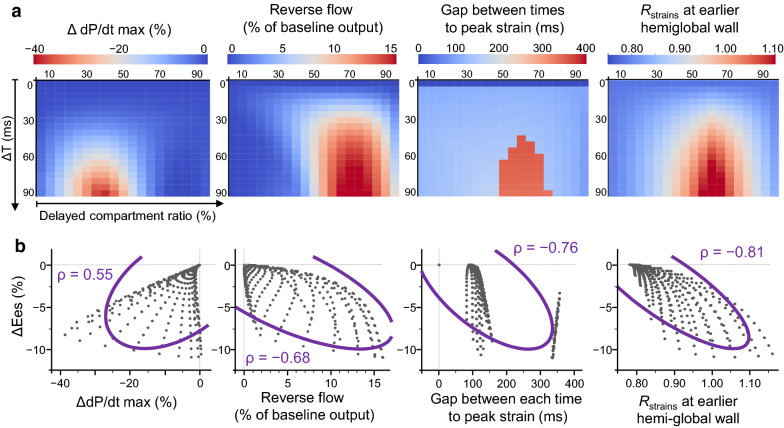
Impact of the ratio of the delayed compartment and an activation delay on simulated hemodynamics. Tiled heat maps colored according to the change in maximal derivatives of ventricular pressure (ΔdP/dt max) relative to its baseline value (Δ*T* = 0), the amount of the reverse flow from the delayed to earlier activated compartment during systole (expressed in percentage of the baseline output), the difference in times to peak strain between earlier and delayed hemiglobal strain curves, and R_strains_ on earlier hemiglobal strain (defined in main text) are shown in **a**. In **b**, their correlation is shown with the relative change in ventricular contractility (ΔEes). Gray dots represent a total of 361 simulated conditions; i.e., 19 conditions of an activation delay of Δ*T* from 0 to 90 ms every 5 ms multiplied by 19 conditions of the ratio of the delayed compartment from 5% to 95% every 5%. The correlation of Δd*P*/d*t* max and reverse flow with ΔEes were heterogeneous. The gap between times to peak of earlier and delayed hemiglobal strain gave nonconsecutive change. The increase in R_strains_ at earlier hemiglobal strain can provide a good prediction of reduced ventricular contractility. Purple curves indicate a 95% confidence ellipse; ρ, Spearman’s rank correlation coefficient

Furthermore, we sought the best predictor of decreased ventricular contractility (negative ΔEes) among the following four simulated hemodynamic parameters: the change in the maximal derivatives of ventricular pressure (ΔdP/dt max) relative to its baseline value (Δ*T* = 0), the amount of reverse flow from the delayed to the earlier activated compartment during systole (expressed in percentage of the baseline output), the difference in time to peak strain between earlier and delayed hemiglobal strain curves, and R_strains_ calculated in the earlier activated hemiglobal strain curve. As shown in Fig. 6, the increase in R_strains_ predicted reduced ventricular contractility.

Clinical characteristics of an uncoordinated wall motion at earlier activated ventricular wall.

**Fig. 7:**
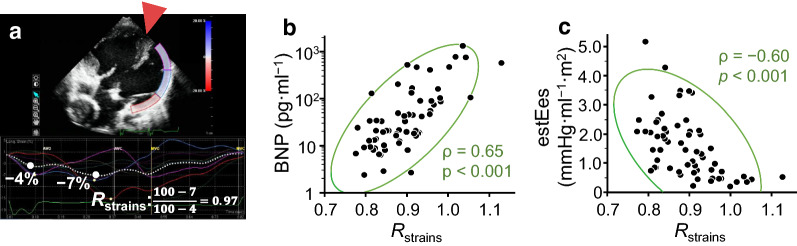
Clinical impact of an uncoordinated wall motion at earlier activated ventricular wall. **a** A representative speckle tracking echocardiogram at three segments applied to earlier activated ventricular wall among seven segments of global longitudinal tracking in a patient with single ventricle. The region of interest did not consider the insertion point of the rudimentary septum (red triangle). Using an obtained strain curve (white dotted line), an uncoordinated wall motion was evaluated by calculating the strain ratio (R_strains_) of two values during the QRS period and the ejection period as an equation at the bottom. **b** Positive correlation between R_strains_ and plasma B-type natriuretic peptide (BNP) level in 62 investigated patients with single ventricle. **c** Negative correlation between R_strains_ and estimated ventricular elastance at end-systole (estEes). Green curves indicate a 95% confidence ellipse; *ρ*, Spearman’s rank correlation coefficient

Finally, we evaluated the local wall strain at earlier activated three segments irrespective of an insertion point of the septum and calculated R_strains_ in the present cohort of patients with single ventricle (Fig. 7). The R_strains_ exhibited a significant positive correlation with the BNP level (*ρ* = 0.65, *p* < 0.001) and a negative association with estEes (*ρ* = − 0.60, *p* < 0.001).

## Discussion

To the best of our knowledge, this is the first study to analyze the detrimental hemodynamic effect of mechanical discoordination, highlighting the importance of the ratio of the delayed wall, AR_delayed_, in addition to an electrical delay. We demonstrated that the ventricular morphology with AR_delayed_ of 60–70% in the patient study and approximately 70% in the simulation study was most vulnerable to a prolonged activation delay, representing an uncoordinated motion at an earlier activated ventricular wall (an increase in R_strains_).

### Morphological characteristics and pathogenesis of mechanical discoordination

The results of the patient study and the computational simulation both identified that the extent of electrical disparity was the primary determinant of ventricular dysfunction and AR_delayed_ was the secondary determinant (Table 2). The most vulnerable ventricular morphology to the electrical disparity is a ventricle in which a delayed wall area accounts for more than a half. Several factors could contribute to the pathogenesis as discussed in the following paragraphs.

In the present simulation, changes in the ventricular volume were proportional to those in the ventricular area. With regard to the ventricular preload, the earlier activated ventricular wall with a relatively small area will lose a larger proportion of its ventricular area during preceding activation compared with the earlier activated ventricular wall with a relatively large area (Fig. 3b). After activation of a delayed wall starts, the earlier activated wall must restore its area (or volume) to balance the contractile force against the delayed wall, resulting in a paradoxical stretch during mid-systole. Besides, since the delayed ventricular wall with a relatively large area will have a theoretically higher distensibility compared with the delayed ventricular wall with a small area, a considerably greater area of the delayed ventricular wall will be stretched before activation, which is analogous to receiving a larger volume from the earlier activated wall. This may induce more inefficient contraction to maintain the same cardiac output.

The results of this study also demonstrated that the ventricle would become insensitive to an electrical delay when a delayed wall area reaches more than 90% (Fig. 4). It is natural because the ventricle presenting delayed activation in almost all ventricular areas is physiologically analogous to the normal ventricle with a prolonged atrioventricular delay. Although the prolonged atrioventricular delay can also decrease cardiac output [23], the negative impact is relatively small.

### Generalization to mechanical discoordination in anatomically normal adults

Our analysis of mechanical discoordination in single-ventricle anatomy provides insight into intraventricular discoordination in adult biventricular physiology. In such patients, the area ratio of a delayed wall will be characterized by the difference in the conduction velocities between two functional conducting systems. As for the patients with LBBB, the septum is rapidly activated by the right bundle branch, whereas the left ventricular (LV) free wall is activated late by a gradually propagating impulse originating from the right ventricle. As for patients with accessory atrioventricular pathways and those under resynchronization therapy (LV pacing), earlier and delayed conductions propagate independently and meet at the middle of the left ventricle. Hence, the ventricle is functionally separated into earlier activated and delayed compartments with various wall area ratios. When the earlier activated compartment is large enough but less than half of the ventricular wall, the ventricle ought to be vulnerable to the widened time difference between two activation onsets. For instance, clinical evidence of patients with the preexcitation syndrome suggests that patients with right-sided septal or paraseptal accessory pathways, but not patients of preexcitation at the LV free wall, suffer from ventricular dysfunction [24, 25]. The preexcitation at the LV free wall conducts slowly and excites only a small ventricular area, whereas the impulse through the septal accessory pathway conducts fast and excites a large area of the basal septum in advance of the activation by the normal conduction system initiating from the apical septum [26], which may contribute to mechanical discoordination.

### Potential clinical implications

As for patients with single-ventricle anatomy, despite diverse anatomical variants, those with a codominant-type single ventricle (like that presented in Fig. 1b) are expected to have a greater risk of cardiac dysfunction associated with prolonged QRS duration. Rosner et al. [27] reported that ventricular dysfunction with an uncoordinated wall motion was frequently observed in patients with two sizable ventricular components, especially with the dominant right ventricle along with the hypoplastic left ventricle and large ventricular septal defect, suggesting anatomical right bundle branch block-induced dyssynchrony. Their finding is consistent with our result (Fig. 2).

In abnormal left ventricle as well as single ventricle without septal defect, a morphological landmark that separates the earlier activated and delayed ventricular walls cannot be identified easily. As demonstrated in Figs. 6 and 7, regardless of the ratio of the delayed wall, an uncoordinated wall motion (an early shortening followed by a systolic rebound stretch), assessed from earlier activated three hemiglobal segments out of the seven global segments, will allow capturing the pathophysiology of mechanical dyssynchrony. This study provides valuable information that paves the way for further investigation and suggests the impact of the morphological factor on the pathogenesis of cardiac dysfunction associated with mechanical discoordination. Moreover, the importance of regional mechanics on efficient cardiac performance can be emphasized in common with other situations such as during a cardiac compression [28].

### Limitations

First, the patient population was small, heterogeneous, and retrospectively analyzed. We focused exclusively on ventricular anatomy among participants with various physiological and surgical backgrounds. Although the cause–effect relationship between the ratio of the delayed wall and ventricular dysfunction was unclear due to subject heterogeneity, the simulation study supported the interpretation that the ventricular morphology contributed to the ventricular dysfunction. Second, the simulated ventricle was designed as a simple hemisphere consisting of two compartments in a zero-dimensional circulatory model, neglecting the distribution of pressure and flow wave dynamics. The real ventricular walls are not spherical, implying that the radii of the curvature along the circumferential and base-to-apex direction are different. Moreover, inhomogeneous myofibril direction of the morphological left and right ventricles, the presence of outflow and rudimentary septum, surgical scar, and local remodeling may interdependently affect cardiac mechanics. A three-dimensional finite element model should be accurate and suited to describe local inhomogeneity in mechanical load for patient-specific modeling. However, such a model may be time-consuming, and its calculation effort is extremely high. Our lumped-parameter model is simple but sufficient enough to represent physiological implications and to successfully mimic clinical data, as shown in Figs. 3c and 5c. Previous studies modeling LBBB-induced dyssynchrony, which lumped the three ventricular wall segments of the right ventricle, the septum, and the LV free wall, also provided qualitative and sometimes quantitative agreement with the clinical measurements [8]. Despite the inherent limitations, our model also provides a clearer mechanistic view. For further studies, the finite element model is required in the future to evaluate possible errors with the applied simplifications of our model. Third, the myocardial contractility of different sized patients in the clinical study (Eq. 1) was roughly calculated based on the ventricular volume normalized for body surface area [29], not for a ventricular mass [30] due to technical limitation. The volume intercept of the end-systolic pressure–volume relation curve was also neglected. Because a ventricular mass and an unstressed ventricular volume are larger in deteriorated hearts than intact hearts [31], the estEes must have been underestimated, especially in patients with ventricular dysfunction. Nevertheless, we believe that the comparison between subjects would still be possible. Even if the true maximum elastance is assumed to be 150% as much as estEes [31], the change is relatively small compared with a wide distribution of estEes in our cohort. Lastly, our simulation assessed only acute mechanical and hemodynamic changes associated with an activation delay. Therefore, our model would not be able to provide quantitative consistency with pathological development. In a chronic phase, regional myocardial remodeling at an earlier activated wall proven in a canine study [32] and human [33] has been shown to influence myocardial mechanics [34]. Reduced mechanical workload due to stretch motion during mid-systole induces the local thinning of an earlier activated myocardial region [35], resulting in an exaggeration of a systolic rebound stretch. Clinical profiles in our cohort may reflect a resultant vicious circle of uncoordinated wall motion and regional adverse remodeling.

## Conclusions

The clinical study on patients with single-ventricle anatomy and the simulation study using the 0D circulatory modeling both suggested that the ventricle with a regional delay in 60–70% of the total ventricle (in area or volume) would be most vulnerable to an activation delay and reveal an uncoordinated motion at an earlier activated wall. The ratio of the delayed ventricular wall had a significant impact on mechanical discoordination associated with an activation delay, potentially highlighting an indicator of cardiac dysfunction. Further study is warranted to confirm this interpretation.

### Supplementary information

**Additional file 1: Appendix A.** Estimation of a wall area ratio. **Appendix B.** Hemodynamic simulation of single-ventricle physiology. **Appendix C.** Strain measurement from the simulation. **Figure S1.** Estimation of a wall area ratio based on a hemispherical ventricular model. **Figure S2.** Hemodynamic simulation with a two-compartment ventricular model after total cavopulmonary connection (Fontan circulation). **Figure S3.** Strain measurement from a two-compartment ventricular model. **Figure S4.** Change in pressure rise depending on an activation delay and the delayed compartment volume ratio.

## Data Availability

The datasets generated and/or analyzed during the current study are available from the corresponding author on reasonable request.

## Abbreviations

AR_delayed_: Area ratio of the delayed wall
BNP: B-type natriuretic peptide
estEes: Estimated ventricular end-systolic elastance
ESVi: End-systolic ventricular volume index
LBBB: Left bundle branch block
LV: Left ventricle
R_strains_: Strain ratio (defined in the manuscript)
