# Supplementary material for: Impact of delayed ventricular wall area ratio on pathophysiology of mechanical dyssynchrony: implication from single-ventricle physiology and 0D modeling
Source: J Physiol Sci. 2020 Aug 6;70:38. doi: 10.1186/s12576-020-00765-y (PMC10716988; doi:10.1186/s12576-020-00765-y)
Supplement: Supplementary file 1 — Additional file 1: Appendix A. Estimation of a wall area ratio. Appendix B. Hemodynamic simulation of single-ventricle physiology. Appendix C. Strain measurement from the simulation. Figure S1. Estimation of a wall area ratio based on a hemispherical ventricular model. Figure S2. Hemodynamic simulation with a two-compartment ventricular model after total cavopulmonary connection (Fontan circulation). Figure S3. Strain measurement from a two-compartment ventricular model. Figure S4. Change in pressure rise depending on an activation delay and the delayed compartment volume ratio. [file 12576_2020_765_MOESM1_ESM.docx]

**Additional material**

**Appendix A.** Estimation of a wall area ratio

From an apical four chamber-like view on echocardiograms in patients with single-ventricle anatomy, the ventricular wall can be separated into two areas by an insertion point of the septum. A length ratio of a part of the ventricular wall to the global wall is calculated as described in the methods and represented in Figure 1. In this section, we discuss the estimation of a wall area ratio using a length ratio from a longitudinal view.

The ventricle is modeled as a hemisphere of radius *r* (Figure S1), and a center, basal points, an apex, and an insertion point of the septum are denoted as *O*, *B* or *B’*, *Ap*, and *S*, respectively. A junctional plane perpendicular to the *B’OB* line (x-axis) through *S* intersects a wall surface (A_part_, colored in red) from the whole surface area (A_whole_, colored in gray). A surface area ratio was calculated using the distance *h* between the junctional plane and point *B* as follows:

|  | $Surface area ratio=\frac{A_{part}}{A_{whole}}=\frac{\pi\cdot r\cdot h}{2\pi\cdot r^{2}}=\frac{h}{2r}$ | (S1) |
| --- | --- | --- |

When an angle *BOS* is denoted as *θ* (radian), a length ratio (L_ratio_) was described as follows:

|  | $L_{ratio}=\frac{circumference [B-S]}{circumference [B-Ap-B^{'}]}= \frac{r\cdot\theta}{r\cdot\pi}=\frac{\theta}{\pi}$ | (S2) |
| --- | --- | --- |

A trigonometric identity of angle *θ* was defined using *Equation (Eq.) S1*.

|  | $\cos\theta=\frac{r-h}{r}= 1-2\cdot(Suface area ratio)$ | (S3) |
| --- | --- | --- |

Rearranging *Eqs. S2* and *S3* yields an estimation of a surface area ratio using L_ratio_ as follows:

|  | $Surface area ratio=\frac{1-cos (\pi\cdot L_{ratio})}{2}$ | (S4) |
| --- | --- | --- |

**Appendix B.** Hemodynamic simulation of single-ventricle physiology

We simulated hemodynamics of single-ventricle physiology in the postoperative state after total cavopulmonary connection. The electrical analog of the cardiovascular system and corresponding schema are shown in Figure S2a and S2b.

Ventricular wall and atrial chamber contractions were characterized by a time-varying elastance theory [1], which describes instantaneous pressure–volume relations [2]:

|  | $P\left( t \right) = \left[ P_{es}\left( V \right)-P_{ed}\left( V \right) \right]\cdot\varepsilon\left( t \right)+P_{ed}\left( V \right)$ | (S5) |
| --- | --- | --- |
|  | $P_{es}\left( V \right) = E_{max}\cdot\left( V(t) - V_{0} \right)$ | (S6) |
|  | $P_{ed}\left( V \right) = A\cdot\left[ exp\left\{ B\cdot\left( V(t)-V_{0} \right) \right\}-1 \right]$ | (S7) |

where P(t) is ventricular pressure as a function of time, P_es_(V) is end-systolic pressure as a function of volume, P_ed_(V) is end-diastolic pressure as a function of volume, ε(t) is normalized elastance as a function of time during a cardiac cycle, E_max_ is maximal elastance, V(t) is ventricular volume as a function of time, V_0_ is the volume axis intercept of the pressure–volume relationship, A is the scaling factor for end-diastolic pressure-volume relationship (EDPVR), and B is the exponent for EDPVR. Since we focused on detailed ventricular behavior, the normalized elastance curve of the ventricle was determined by cubic spline interpolation of experimental data in canines and humans [3,4] like a skewed sine curve, as shown in Figure S2c. For atrial chambers, a cosine curve was adopted as the normalized elastance:

|  | $\varepsilon\left( t \right)=0.5\cdot\left[ 1 - \cos\left( \frac{\pi}{T_{max}} t \right) \right] (0\leq t<2 T_{max})$ | (S8) |
| --- | --- | --- |
|  | $\varepsilon\left( t \right) = 0 (2 T_{max}\leq t)$ | (S9) |

where T_max_ represents the time to maximal elastance.

To simulate an activation delay at the local ventricular wall, we designated time delay in activation onset at a part of the ventricular wall by ΔT (ms). We divided the ventricle into 20 and introduced an activation delay in *i*/20 of the whole ventricular volume (*i*: an integer, 1 ≤ *i* ≤ 19). Our analysis is thus the same as a two-compartment model (earlier activated [normal] vs delayed); i.e., the volume of the delayed compartment ranged from 5% to 95% of the total end-diastolic ventricular volume in steps of 5%, and correspondingly that of the earlier activated (normal) compartment ranged from 95% to 5% of the total end-diastolic ventricular volume. Each ventricular volume can be represented by volume per unit (= 5% of the total end-diastolic volume) as follows:

|  | $V_{1}(t)=v_{1}(t)\cdot(20-i), V_{2}(t)=v_{2}(t)\cdot i$ | (S10) |
| --- | --- | --- |

where V_1_(t) and V_2_(t) represent the ventricular volume in the earlier and delayed compartments as a function of time, respectively; and v_1_(t) and v_2_(t) are volumes per unit of earlier and delayed compartments as a function of time, respectively. Assuming that each compartment has each homogeneous intracavity pressure, *Eq. S5–7* can be rewritten as follows:

|  | $P_{1}\left( t \right) = \left[ P_{es}\left( V_{1} \right)-P_{ed}\left( V_{1} \right) \right]\cdot\varepsilon\left( t \right)+P_{ed}\left( V_{1} \right)$ | (S11) |
| --- | --- | --- |
|  | $P_{es}\left( V_{1} \right)= E_{max}\cdot\left( v_{1}\left( t \right)- v_{0} \right)$ | (S12) |
|  | $P_{ed}\left( V_{1} \right) = A\cdot\left[ exp\left\{ B\cdot\left( v_{1}\left( t \right)-v_{0} \right) \right\}-1 \right]$ | (S13) |
|  | $P_{2}\left( t \right) = \left[ P_{es}\left( V_{2} \right)-P_{ed}\left( V_{2} \right) \right]\cdot\varepsilon\left( t-\Delta T \right)+P_{ed}\left( V_{2} \right)$ | (S14) |
|  | $P_{es}\left( V_{2} \right)= E_{max}\cdot\left( v_{2}\left( t \right)- v_{0} \right)$ | (S15) |
|  | $P_{ed}\left( V_{2} \right) = A\cdot\left[ exp\left\{ B\cdot\left( v_{2}\left( t \right)-v_{0} \right) \right\}-1 \right]$ | (S16) |

where P_1_(t) and P_2_(t) represent the ventricular pressure in the earlier and delayed compartments as a function of time, respectively, and v_0_ is the volume axis intercept per unit of the pressure–volume relationship. P_1_(t) and P_2_(t) are almost identical when earlier and delayed compartments are connected with a low ohm resistor (R_intra_, Figure S2a). The v_0_ was assigned to be 15% of the volume per unit at end-diastole [3] to accomplish the appropriate estimation of ejection fraction and myocardial strain. As a result, v_1_(t) and v_2_(t) are identical during late diastole because each normalized elastance becomes zero, but are different during systole due to intraventricular communication involving different activation onset, resulting in mechanical dyssynchrony.

Systemic and pulmonary circulations were constructed based on a three-element Windkessel model, which consisted of a proximal characteristic impedance (Z), a lumped vascular capacitance (C), and a lumped peripheral arterial resistance (R) [5]. Systemic and pulmonary circuits were connected in series for modeling postoperative circulation after total cavopulmonary connection (Fontan operation) with an extracardiac conduit.

The heart valves permit flow only in one direction. The pressure–flow relationship across an outlet (semilunar) valve was assumed to be a simple resistor. The pressure drop across the atrioventricular valve is dominated by Bernoulli’s law [6], which is given by:

|  | $P_{atrium}- P_{ventricle} \left( P_{atrium}>P_{ventricle} \right)$  $= \frac{1.06}{2\cdot1,333\cdot{{Area}_{avv}}^{2}}\cdot Q\cdot\left\vert Q \right\vert+ R\cdot Q + L\cdot\frac{dQ}{dt}$ | (S17) |
| --- | --- | --- |
|  | $Q = 0 (P_{atrium}\leq P_{ventricle})$ | (S18) |

where P_atrium_ is the atrial pressure, P_ventricle_ is the ventricular pressure, Area_avv_ is the antegrade effective orifice area of the atrioventricular valve when the atrial pressure exceeds the ventricular pressure, Q is the flow across the valve, R is the viscous resistance, and L is the inertial inductance. Viscous resistance (R) was assumed to be small and set to zero.

Blood volume is the sum of functional two pools: unstressed blood volume and stressed blood volume (V_stressed_). An increase in V_stressed_ linearly increases the pressure at each vascular compartment according to the compliance (C):

|  | $P = \frac{V_{stressed}}{C}$ | (S19) |
| --- | --- | --- |

The total stressed blood volume equals to the sum of V_stressed_ in all compartments [7]. The change in volume in each compartment is calculated from the difference of instantaneous blood inflow and outflow.

The cardiac chamber consisted of a ventricle with two compartments and an atrium, which were accompanied by an atrioventricular valve connected to both ventricular compartments and by an aortic valve associated with the delayed compartment. The value of each parameter of our model was set to be appropriate for a 40-kg body (body surface area of 1.35 m^2^), and the heart rate was fixed at 80 beats per minute in concordance with the subjects in our patient study. Hemodynamic parameters in Table 3 and baseline characteristics (ΔT = 0 ms) in Table 4 were derived from the clinical data of our institute [8] and were comparable to the patient study.

An atrioventricular interval is fixed, whereas the activation of the delayed compartment starts later from that of the other by 0–90 ms every 5 ms. The 90-ms delay corresponds to 10 standard deviations of QRS elongation in our patient study.

To evaluate ventricular contractility, the elastance of the whole ventricle was monitored as follows:

|  | $E_{1}(t)= \frac{P_{1}(t)}{[v_{1}\left( t \right)-v_{0}]\cdot(20-i)}$ | (S20) |
| --- | --- | --- |
|  | $E_{2}(t)= \frac{P_{2}(t)}{[v_{2}\left( t \right)-v_{0}]\cdot i}$ | (S21) |
|  | $\frac{1}{E\left( t \right)}= \frac{1}{E_{1}\left( t \right)}+\frac{1}{E_{2}\left( t \right)}$ | (S22) |

where E_1_(t), E_2_(t), and E(t) represent the elastance of the earlier activated compartment, delayed compartment, and the global ventricle as a function of time, respectively. The maximum value of E(t) during the cardiac cycle represented the contractility of the whole ventricle and was denoted as Ees.

**Appendix C.** Strain measurement from the simulation

In this section, we will discuss how to calculate a ventricular strain based on our hemodynamic modeling.

A ventricle is designed as a hemisphere whose radius r(t) continuously changes as a function of time during a cardiac cycle (Figure S3). A longitudinal strain is a deformation of a semicircle B_1_–B_2_ (black line in Panel a) on the hemisphere surface in a plane y = 0 (schematized in Panel d1 and d2). A global longitudinal strain is evaluated using a seven-segment model, but the apical cap should not be included to assess regional strain according to the recommendation [9]. We estimated two hemiglobal longitudinal strains: a hemiglobal strain at three segments on the earlier activated side [hGLS_earlier_(t), %] that measures a local deformation between a side of the apical cap (point *ApC_1_*) and a lateral basal point (point *B_1_*) (brown lines) relative to a reference length at end-diastole (t = 0), and a hemiglobal strain at the other three segments on the delayed side [hGLS_delayed_(t), %] that measures a local deformation between the other side of the apical cap (point *ApC_2_*) and the other lateral basal point (point *B_2_*) (purple lines) relative to a reference length at end-diastole (t = 0). Points *ApC_1_* and *ApC_2_* internally divide a semicircle B_1_–B_2_ by 3:4 and 4:3, respectively.

A hGLS_earlier_(t) is defined as follows:

|  | $\mathrm{hGLS}_{earlier}\left( t \right)=\frac{r\left( t \right)\cdot\varphi\left( t \right)-r\left( 0 \right)\cdot\varphi\left( 0 \right)}{r\left( 0 \right)\cdot\varphi\left( 0 \right)}\cdot100$ | (S22) |
| --- | --- | --- |

where φ(t) represents an angle *B_1_OApC_1_* as a function of time. With φ(0) = 3π/7, *Eq. S22* can be rewritten as follows:

|  | $\mathrm{hGLS}_{earlier}\left( t \right)=\frac{700}{3\pi}\cdot\varphi(t)\cdot\frac{r\left( t \right)}{r\left( 0 \right)}-100$ | (S23) |
| --- | --- | --- |

As previously described in Appendix B, two volumetric values in the respective ventricular compartments are obtained in our simulation as V_1_(t) and V_2_(t). By focusing on the change in the total volume, we can obtain a radius ratio of the hemisphere-model ventricle as follows:

|  | $\frac{r\left( t \right)}{r\left( 0 \right)}=\sqrt[3]{\frac{V_{1}\left( t \right)+V_{2}\left( t \right)}{V_{1}\left( 0 \right)+V_{2}\left( 0 \right)}}$ | (S24) |
| --- | --- | --- |

A junction semicircle (red lines) on the hemisphere surface that shifts in perpendicular to the x-axis separates a wall surface into two areas as A_1_(t) (colored in yellow) and A_2_(t) (colored in blue). Because the curvature of each ventricular wall surface is identical as a reciprocal of radius r(t) as shown in Panel b, the relationship between the volume and surface area of each compartment can be described as follows:

|  | $V_{1}(t)=\frac{1}{3}\cdot A_{1}(t)\cdot r(t)$ | (S25) |
| --- | --- | --- |
|  | $V_{2}(t)=\frac{1}{3}\cdot A_{2}(t)\cdot r(t)$ | (S26) |
|  | ${V_{1}\left( t \right)+V}_{2}(t)=\frac{2}{3}\cdot\pi\cdot{r(t)}^{3}$ | (S27) |

Dividing *Eq. S25* by *Eq. S27* or dividing *Eq. S26* by *Eq. S27* gives

|  | $A_{1}(t)=2\pi\cdot{r(t)}^{2}\cdot\frac{V_{1}\left( t \right)}{V_{1}\left( t \right)+V_{2}\left( t \right)}$ | (S28) |
| --- | --- | --- |
|  | $A_{2}(t)=2\pi\cdot{r(t)}^{2}\cdot\frac{V_{2}\left( t \right)}{V_{1}\left( t \right)+V_{2}\left( t \right)}$ | (S29) |

The apical cap is shifted laterally during a cardiac cycle (this lateral apical motion is clinically called “apical rocking”). Because a surface area of each compartment uniformly contracts or dilates, a semicircle line perpendicular to the x-axis through *ApC_1_* (green lines) separates the corresponding surface area at the same ratio throughout a cardiac cycle. When *ApC_1_* is on surface 1 (shown in Panel d1), a surface area ratio of the area on the *B_1_* side to the surface area 1 is given using an angle φ(t) as follows:

|  | $\frac{\pi\cdot r(0)\cdot\left[ r(0)-r(0)\cdot\cos\varphi(0) \right]}{A_{1}(0)}=\frac{\pi\cdot r(t)\cdot\left[ r(t)-r(t)\cdot\cos\varphi(t) \right]}{A_{1}(t)}$ | (S30) |
| --- | --- | --- |

With cos φ(0) ≈ 0.223, applying *Eq. S28* to *Eq. S30* yields

|  | $\cos\varphi(t)=1-0.777\cdot\frac{V_{1}\left( 0 \right)+V_{2}\left( 0 \right)}{V_{1}\left( 0 \right)}\cdot\frac{V_{1}\left( t \right)}{V_{1}\left( t \right)+V_{2}\left( t \right)}$ | (S31) |
| --- | --- | --- |

Note that when *ApC_1_* is on surface 1, the left-hand side of *Eq. S30* is under 1.

|  | $\frac{\pi\cdot r(0)\cdot\left[ r(0)-r(0)\cdot\cos\varphi(0) \right]}{A_{1}(0)}<1$ | (S32) |
| --- | --- | --- |

Applying *Eq. S29* to *Eq. S32* yields

|  | $\frac{V_{2}\left( 0 \right)}{V_{1}\left( 0 \right)+V_{2}\left( 0 \right)}<\frac{1+\cos\varphi(0)}{2}\approx0.61$ | (S33) |
| --- | --- | --- |

When *ApC_1_* is on surface 2 (shown in Panel d2), a surface area ratio of the area on the *B_2_* side to the surface area 2 was given using angle φ(t) as follows:

|  | $\frac{\pi\cdot r(0)\cdot\left[ r\left( 0 \right)+r(0)\cdot\cos\varphi(0) \right]}{A_{2}(0)}=\frac{\pi\cdot r(t)\cdot\left[ r\left( t \right)+r(t)\cdot\cos\varphi(t) \right]}{A_{2}(t)}$ | (S34) |
| --- | --- | --- |

With cos φ(0) ≈ 0.223, applying *Eq. S29* to *Eq. S34* yields

|  | $\cos\varphi(t)=-1+1.223\cdot\frac{V_{1}\left( 0 \right)+V_{2}\left( 0 \right)}{V_{2}\left( 0 \right)}\cdot\frac{V_{2}\left( t \right)}{V_{1}\left( t \right)+V_{2}\left( t \right)}$ | (S35) |
| --- | --- | --- |

If cos φ(t) is denoted as K(t), applying *Eqs. S23*, *S31*, and *S33* to *Eq. S23* finally gives

|  | $\mathrm{hGLS}_{earlier}\left( t \right)=\frac{700}{3\pi}\cdot\arccos K(t)\cdot\sqrt[3]{\frac{V_{1}\left( t \right)+V_{2}\left( t \right)}{V_{1}\left( 0 \right)+V_{2}\left( 0 \right)}}-100$ | (S36) |
| --- | --- | --- |

where

|  | $K\left( t \right)=1-0.777\cdot\frac{V_{1}\left( 0 \right)+V_{2}\left( 0 \right)}{V_{1}\left( 0 \right)}\cdot\frac{V_{1}\left( t \right)}{V_{1}\left( t \right)+V_{2}\left( t \right)}$  $\left( when \frac{V_{2}\left( 0 \right)}{V_{1}\left( 0 \right)+V_{2}\left( 0 \right)}<0.61 \right)$ | (S37) |
| --- | --- | --- |
|  | $K\left( t \right)=-1+1.223\cdot\frac{V_{1}\left( 0 \right)+V_{2}\left( 0 \right)}{V_{2}\left( 0 \right)}\cdot\frac{V_{2}\left( t \right)}{V_{1}\left( t \right)+V_{2}\left( t \right)}$  $\left( when \frac{V_{2}\left( 0 \right)}{V_{1}\left( 0 \right)+V_{2}\left( 0 \right)}\geq0.61 \right)$ | (S38) |

As for hGLS_delayed_(t), the calculation is the same as a mirror image for hGLS_earlier_(t). Inverting subscript 1 and 2 in *Eq. S36, S37*, and *S38* yields

|  | $\mathrm{hGLS}_{delayed}\left( t \right)=\frac{700}{3\pi}\cdot\arccos J(t)\cdot\sqrt[3]{\frac{V_{1}\left( t \right)+V_{2}\left( t \right)}{V_{1}\left( 0 \right)+V_{2}\left( 0 \right)}}-100$ | (S39) |
| --- | --- | --- |

where

|  | $J\left( t \right)=1-0.777\cdot\frac{V_{1}\left( 0 \right)+V_{2}\left( 0 \right)}{V_{2}\left( 0 \right)}\cdot\frac{V_{2}\left( t \right)}{V_{1}\left( t \right)+V_{2}\left( t \right)}$  $\left( when \frac{V_{2}\left( 0 \right)}{V_{1}\left( 0 \right)+V_{2}\left( 0 \right)}>0.39 \right)$ | (S40) |
| --- | --- | --- |
|  | $J\left( t \right)=-1+1.223\cdot\frac{V_{1}\left( 0 \right)+V_{2}\left( 0 \right)}{V_{1}\left( 0 \right)}\cdot\frac{V_{1}\left( t \right)}{V_{1}\left( t \right)+V_{2}\left( t \right)}$  $\left( when \frac{V_{2}\left( 0 \right)}{V_{1}\left( 0 \right)+V_{2}\left( 0 \right)}\leq0.39 \right)$ | (S41) |

To evaluate an uncoordinated motion of earlier activated ventricular wall, a strain ratio (*R*_strains_) was calculated as follows:

|  | $R_{strains}=\frac{100+{\mathrm{hGLS}_{earlier}(t}_{1})}{100+{\mathrm{hGLS}_{earlier}(t}_{2})}$ | (S42) |
| --- | --- | --- |

where hGLS_earlier_(t_1_) is the minimum value of hGLS_earlier_(t) between atrioventricular valve closure and aortic valve opening (isovolumic contraction period) and hGLS_earlier_(t_2_) is the minimum nadir value of hGLS_earlier_(t) between aortic valve opening and closure (ejection period). This index is larger if an earlier activated ventricular wall is forcedly dilated and fails to shorten during delayed ventricular wall contraction.

References

1. Suga H, Sagawa K (1974) Instantaneous pressure-volume relationships and their ratio in the excised, supported canine left ventricle. Circ Res 35:117-126

2. Burkhoff D, Tyberg JV (1993) Why does pulmonary venous pressure rise after onset of LV dysfunction: a theoretical analysis. Am J Physiol 265:H1819-1828

3. Suga H, Sagawa K, Shoukas AA (1973) Load independence of the instantaneous pressure-volume ratio of the canine left ventricle and effects of epinephrine and heart rate on the ratio. Circ Res 32:314-322

4. Senzaki H, Chen CH, Kass DA (1996) Single-beat estimation of end-systolic pressure-volume relation in humans. A new method with the potential for noninvasive application. Circulation 94:2497-2506

5. Westerhof N, Elzinga G, Sipkema P (1971) An artificial arterial system for pumping hearts. J Appl Physiol 31:776-781

6. Sun Y, Sjoberg BJ, Ask P, Loyd D, Wranne B (1995) Mathematical model that characterizes transmitral and pulmonary venous flow velocity patterns. Am J Physiol 268:H476-489

7. Guyton AC, Lindsey AW, Abernathy B, Richardson T (1957) Venous return at various right atrial pressures and the normal venous return curve. Am J Physiol 189:609-615

8. Ohuchi H, Miyazaki A, Wakisaka Y, Watanabe K, Kishiki K, Yamada O, Yagihara T, Echigo S (2009) Systemic ventricular morphology-associated increased QRS duration compromises the ventricular mechano-electrical and energetic properties long-term after the Fontan operation. Int J Cardiol 133:371-380

9. Lang RM, Badano LP, Mor-Avi V, Afilalo J, Armstrong A, Ernande L, Flachskampf FA, Foster E, Goldstein SA, Kuznetsova T, Lancellotti P, Muraru D, Picard MH, Rietzschel ER, Rudski L, Spencer KT, Tsang W, Voigt JU (2015) Recommendations for cardiac chamber quantification by echocardiography in adults: an update from the American Society of Echocardiography and the European Association of Cardiovascular Imaging. J Am Soc Echocardiogr 28:1-39 e14

Figure legends

**Figure S1.** Estimation of a wall area ratio based on a hemispherical ventricular model.

The schemata represent a ventricle modeled as a hemisphere (Panel a), its short-axis view (cut plane: z = 0, Panel b), and its longitudinal view (cut plane: y = 0, Panel c). A center, basal points, an apex, and an insertion point of the septum are denoted as *O*, *B* or *B’*, *Ap*, and *S*, respectively. A junction plane, which represents an insertion point of the septum perpendicular to the x-axis through *S*, intersects a wall surface (A_part_, colored in red) from the whole surface area (A_whole_, colored in gray). An angle *BOS* is denoted as θ (radian), and a length ratio (L_ratio_) is a ratio of circumference B–S (purple double arrow) to B–Ap–B’ (black double arrow).

**Figure S2.** Hemodynamic simulation with a two-compartment ventricular model after total cavopulmonary connection (Fontan circulation).

a. An electrical analog of the simulated circulation with a lumped parameter model. A heart was modeled with an atrium and a ventricle with two compartments as time-varying elastic chambers, an intraventricular communication as a resistor (R_intra_), an atrioventricular valve (AVV), and an aortic valve as diodes. Systemic and pulmonary circulations of the three-element Windkessel model are directly connected in series with a conduit pathway (after the Fontan operation). Z, characteristic impedance; C, compliance; R, resistance; AoV, aortic valve; ao, aorta; sa, systemic arterial vasculature; sv, systemic venous vasculature; pa, pulmonary arterial vasculature; pv, pulmonary venous vasculature.

b. Schema of postoperative Fontan circulation. The green pathway represents an extracardiac conduit after total cavopulmonary connection. Yellow and blue dotted double arrows represent earlier and delayed activated ventricular walls, respectively.

c. The normalized elastance curve of the ventricle. Since we focused on detailed ventricular behavior, the normalized elastance curve of the ventricle (red line, ε(t)) was determined by cubic spline interpolation of experimental data (blue open circles) in canines and humans like a skewed sine curve. T_max_, time to maximal elastance.

**Figure S3.** Strain measurement from a two-compartment ventricular model.

The schemata represent a hemispherical ventricular model (Panel a and b), its short-axis view (cut plane: z = 0, Panel c), and its longitudinal view (cut plane: y = 0, Panel d). A center, basal points, and two sides of an apical cap (the black circle in Panel a) are denoted as *O*, *B_1_*, *B_2_*, *ApC_1_*, and *ApC_2_*, respectively. A junction semicircle (red lines) perpendicular to the x-axis divides a wall surface into two walls (colored in yellow and blue). According to the area ratio, two patterns are schematized in Panel d1 and d2. The radius of the hemisphere, an angle *B_1_OApC_1_*, a surface area including point *B_1_*, the other area including point *B_2_*, and corresponding volumes (Panel b) as a function of time can be designated as r(t), φ(t), A_1_(t), A_2_(t), V_1_(t), and V_2_(t), respectively. The junction semicircle, *ApC_1_*, and *ApC_2_* are parallel-shifted during a cardiac cycle along the x-axis direction. A longitudinal strain (e.g., brown and purple double-arrow lines) measures a local deformation between two points of interest on a semicircle B_1_–B_2_ (black lines) in a plane y = 0 relative to a reference length at end-diastole (t = 0).

**Figure S4.** Change in pressure rise depending on an activation delay and the delayed compartment volume ratio.

Simulated pressure waveforms are displayed in Panel a. Black and red lines, or yellow and blue vertical lines represent simulated ventricular and arterial pressures, or contraction onsets of the earlier and delayed compartments, respectively. Both the activation delay by ΔT (top to bottom row) and the ratio of the delayed compartment (left to right column) affect ventricular pressure waveforms. Compared with the other conditions, prominent distortion of ventricular pressure rise just after the contraction onset (black triangles) can be found when the delayed compartment had a relatively large volume ratio and a large activation delay. A similar pattern of pressure rise can be speculated from the atrioventricular valve regurgitant Doppler signal in a patient with a codominant-type single ventricle (white triangle, Panel b) just after the onset of systole.
